# Diamond-Like Carbon: A Surface for Extreme, High-Wear Environments

**DOI:** 10.1021/acs.langmuir.3c01438

**Published:** 2023-12-19

**Authors:** N. Sharifi, H. Smith, D. Madden, T. Kehoe, G. Wu, L. Yang, R. J. L. Welbourn, E. G Fernandez, S. M. Clarke

**Affiliations:** † Institute for Energy and Environmental Flows and Yusuf Hamied Department of Chemistry, University of Cambridge, Cambridge CB2 1EW, U.K.; ‡ Institute of Functional Surfaces, School of Mechanical Engineering, University of Leeds, Leeds LS2 9JT, U.K.; § Rutherford Appleton Laboratory, STFC, Chilton, ISIS Neutron & Muon Source, Didcot, Oxon OX11 0QX, U.K.; ∥ XMaS/BM28-ESRF, 71 Avenue Des Martyrs, F-38043 Grenoble, Cedex, France; ⊥ Department of Physics, University of Warwick, Gibbet Hill Road, Coventry CV4 7AL, U.K.

## Abstract

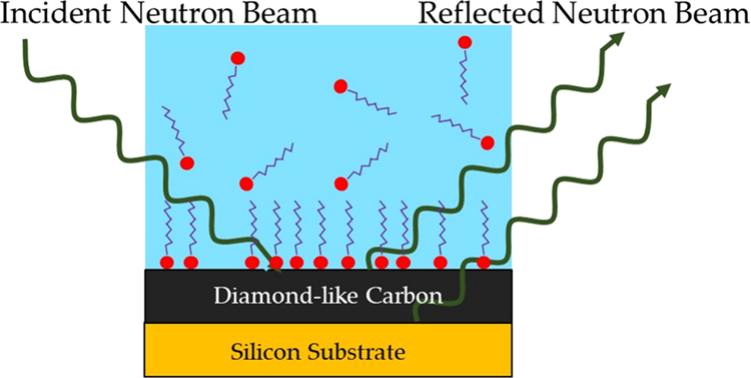

In this study, we present an in-depth characterization of a diamond-like carbon (DLC) film, using a range of techniques to understand the structure and chemistry of the film both in the interior and particularly at the DLC/air surface and DLC/liquid interface. The DLC film is found to be a combination of sp^2^ and sp^3^ carbon, with significant oxygen present at the surface. The oxygen seems to be present as OH groups, making the DLC somewhat hydrophilic. Quartz-Crystal Microbalance (QCM) isotherms and complementary neutron reflectivity data indicate significant adsorption of a model additive, bis(2-ethylhexyl) sulfosuccinate sodium salt (AOT) surfactant, onto the DLC from water solutions and indicate the adsorbed film is a bilayer. This initial study of the structure and composition of a model surfactant is intended to give a clearer insight into how DLC and additives function as antiwear systems.

## Introduction

Many commercial systems involve rubbing surfaces. Under high loads this can lead to wear and failure. For example, the increasing size of wind turbines leads to high loads, of the order of GPa.^1^ In particular, friction between moving parts in the gearbox may lead to damage and high repair costs and downtime,^2,3^ especially where devices are inaccessible offshore. To prevent life-threatening failures of key infrastructure, such as safety valves, some surfaces are coated with wear-resistant layers such as diamond-like-carbon (DLC) coatings. These coatings are used in parallel with molecular additives, which are proposed to adsorb on the DLC to reduce friction and help prevent wear. Previous studies on DLC have focused on tribological measurements.^4−7^ There is also some interest in biological systems such as protein and amino acid adsorption on fluorine-doped DLC.^8,9^ There are a number of studies reported in the literature that have focused on the characterization of hydrogenated amorphous carbon;^10−15^ however, there have been considerably fewer studies on non-hydrogenated DLC.

DLC films are reported to have a complex structure. Diamond has sp^3^ carbons with a crystalline structure. By contrast, graphite has layers of sp^2^ hybridized carbons. The bulk structure of DLC is reported to be an amorphous mixture of both sp^2^ and sp^3^ carbon, with the proportion of sp^2^ and sp^3^ carbons important in determining the properties of the DLC, such as its mechanical hardness and chemical inertness.^16−19^ The amount of sp^2^ and sp^3^ can be partly controlled by the preparation conditions.^16,20,21^ The surface chemistry of the DLC is expected to control the interaction with any molecular additives. However, this is not well reported nor understood. There have been neutron reflectivity (NR) studies that have successfully characterized the DLC film, but the surface chemistry, particularly the adsorption of molecules onto the surface, has not been explored with neutron reflectivity.^10^ There have been reported studies in the literature that aimed to understand the adsorption behavior of additives onto DLC, including XPS analysis and surface friction measurements, but these do not provide quantitative or structural information about the surface adsorbed layers^22^

The surface chemistry of the DLC is important, possibly different from the bulk in composition, and determines the interaction with any molecular additives. In this work, we present an in-depth characterization of a particular representative example of a DLC film (deposited on silicon), using a range of different techniques to gain key insight into the interior structure and particularly into the surface chemistry. To understand the adsorption behavior of additives onto the film surface, we have studied the adsorption of a common representative surfactant, dioctyl sulfosuccinate sodium salt, AOT with Quartz Crystal Microbalance (QCM) and neutron reflectivity measurements, to determine the adsorbed amount and adsorbed layer structure, respectively.

## Experimental Section

### Materials

Deuterium oxide (D_2_O) was obtained from Sigma (>99% purity, >99.9% D atom). Ultrapure water (UPW18.2 MΩ cm) was obtained using a Millipore water purification system. Dioctyl sulfosuccinate sodium salt, NaAOT, was obtained from Sigma (>99% purity).

### DLC Substrates

The DLC films used for QCM studies were purchased from Q-Sense and used as received. The DLC films for neutron reflection were prepared by sputtering onto polished silicon substrates. Several DLC film thicknesses were prepared, as detailed below, between 30 and 300 Å. The silicon substrates used for neutron reflection experiments were 10 cm × 5 cm × 1 cm, and those used for other characterization experiments were 2” diameter wafers (0.5 mm thick), obtained from Crystran. These small silicon substrates are expected to have very similar physical and chemical properties to the larger silicon blocks. The DLC deposition was carried out at the Institute of Functional Surfaces, School of Mechanical Engineering, University of Leeds by means of reactive magnetron sputtering using an industrial-scale Hauzer Flexicoat 850 system to deposit the taC (tetrahedral carbon) thin film. One carbon target (600 mm × 125 mm × 12 mm, 99.995% purity) was installed in the vacuum chamber as the sputtering target. The chamber was then pumped down to reach a base pressure of 1.2 × 10^–6^ mbar, and the substrates were etched by Ar ions for 30 min with a plasma source, to remove residual contamination on their surfaces. Subsequently, the taC film was produced directly on the substrates with high-power impulse magnetron sputtering (HiPIMS). The parameters utilized on the HiPIMS power supply (Huettinger Co.) were: 4 kW average power, 1200 V peak voltage, 1400 A peak current, 60 Hz pulse frequency, and pulse duration of 200 μs. The vacuum pressure was kept at 3 × 10^–3^ mbar with an Ar flow rate of 130 cm^3^/min, and temperature of 310 K. The deposition time was 200 s, with no bias being applied.

For all experiments, the DLC surfaces were cleaned with a Decon 90 solution overnight, thoroughly rinsed with ultrapure water and dried with nitrogen gas. Other cleaning procedures, including soaking in cyclohexane or washing with concentrated nitric acid, were investigated and the resulting surfaces were characterized with contact angle measurements and X-ray photoelectron spectroscopy. The contact angle of 55° (±3) and XPS data were approximately independent of the surface cleaning procedure. Therefore, the Decon-method followed by thorough rinsing was used for all subsequent experiments.

### X-ray Photoelectron Spectroscopy

XPS analysis was performed at HarwellXPS facility using a Thermo NEXSA spectrometer fitted with a monochromated Al kα X-ray source (1486.7 eV), a spherical sector analyzer and 3 multichannel resistive plate, 128 channel delay line detectors. All data were recorded at 19.2 W, with an X-ray beam size of 400 μm × 200 μm. Survey scans were recorded at a pass energy of 200 eV, and high-resolution scans at a pass energy of 40 eV. As the surface DLC layer is likely to have some insulating properties, and charge build-up at the surface can give rise to shifts in the binding energies of the recorded spectra, electronic charge neutralization was achieved using a dual-beam low-energy electron/ion source (Thermo Scientific FG-03), with an ion gun current of 150 μA and ion gun voltage of 45 V. All sample data were recorded at a pressure below 8^–10^ Torr and at a room temperature of 294 K. Angle-resolved measurements were performed at 0, 11.25, 22.5, 33.75, and 45°. Data were analyzed using CasaXPS v2.3.19PR1.0. Peaks were fit with a Shirley background prior to component analysis. Lorentzian symmetric lineshapes, LA (1.53,243), were used to fit components. The fitting process will be discussed in more detail below. The energy is internally referenced in the usual way by comparison with the C (1s) peak at 285.0 eV from the adventitious carbon that is always present on the surface.^23,24^ It is important to note that the analysis presented does not deconvolute the contribution of the adventitious carbon from the DLC coating, however, as subsequent depth profiling shows no significant variation in sp^2^/sp^3^ composition, the adventitious carbon at the surface initially does not have any material effect on the data interpretation.

### Neutron Reflectivity

NR data were collected using the OFFSPEC instrument at the ISIS neutron facility, Rutherford Appleton Laboratory, U.K. Full details of the instrument may be found elsewhere.^25−27^ Specular reflection data were collected at incident angles of 0.5, 1.0, and 2.3°. Silicon substrates coated with DLC were cleaned as specified above before being mounted in a custom-made aluminum cell with a PTFE trough. The beam footprint on the substrate was controlled to illuminate only the surface region within the trough and the collimation was also used to maintain a constant d*q*/*q* resolution throughout the experiment; *q* is the momentum transfer vector defined as
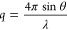
where θ and λ are the beam angle of incidence parallel to the surface and the beam wavelength, respectively. The bare substrates were characterized in D_2_O and H_2_O, the cell was subsequently filled with the surfactant solution of interest using a syringe, and a full set of data, measured at the 3 angles, was stitched together (with a total measurement time of 2 h). Fitting of the NR data was executed using GenX 2.0.0 software.^28^

### X-ray Reflectivity (XRR)

XRR data were collected at the Cavendish Laboratory, Cambridge, using a Bruker D8 X-ray diffractometer with a copper target (wavelength 1.54 Å) and a Goebel mirror. An accelerating voltage of 50 kV and primary beam size of 0.1 mm were used. 0.35 mm Soller slits were inserted before the detector, which was operated in 1D mode.

### Grazing-Incidence Wide-Angle X-ray Scattering (GIWAXS)

The GIWAXS patterns were collected at BM28-XMaS beamline, at the European Synchrotron (Grenoble, France) using a Pilatus 1 M detector, by Dectris. The detector was placed at a distance of 259 mm from the sample. The energy was set at an energy of 12.4 keV (wavelength of 0.1 nm) and the sample was exposed to the beam for 30 s, at angles between 0.06 and 0.2°.

### Time-of-Flight Secondary-Ion Mass Spectrometry (TOF-SIMS)

TOF-SIMS was carried out using the IONTOF TOF-SIMS V instrument at the Department of Materials, Imperial College London. A dual beam arrangement was used with a voltage of 1 kV at 500 eV.^29^

### Quartz Crystal Microbalance

A QCM with a dissipation monitoring, Q-Sense E4 system, from Q-Sense, Sweden, was used for the in situ adsorption of AOT from aqueous solution onto DLC coated sensors. Experiments were performed at the Nanoscience Centre, University of Cambridge. The Q-Sense E4 includes four sensors that can be used in a parallel configuration with temperature control. Full details of the technique and instrument can be found elsewhere.^30^ In brief, the principle behind this technique is that the resonant frequency of the sensor depends on the mass. Therefore, when surfactants adsorb onto the surface of the crystal, the resonant frequency shifts (decreases with increasing mass), which is detected. The decrease in frequency (Δ*f*) can be related to the mass adsorbed (*m*) through the Sauerbrey equation^31^
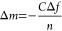
where *C* is a constant equal to 17.7 ng cm^–2^ Hz^–1^ for a quartz crystal, and n is the overtone number (1, 3, 5, ..., 13).

DLC sensors were cleaned by sonicating in 99% ethanol (30 min), sonicated in ultrapure water (30 min), rinsed thoroughly with ultrapure water, and dried with N_2_. The QCM instrument was cleaned with flowing 99% ethanol (30 min) and rinsed thoroughly with ultrapure water (30 min) before the sensors were loaded. Dissolved gases in surfactant solutions were removed by sonication. Ultrapure water was then flowed through (50 μL/min) for 2 h until the frequency of the sensors was stable. Afterward, the lowest surfactant concentration was pumped until a plateau value of the frequency was attained. The next (higher) surfactant concentration was then pumped onto the same crystal, without stopping the device or cleaning the crystal, until a new equilibrium was reached. This was repeated until the whole range of AOT concentrations of interest was completed. The crystal was then flushed with ultrapure water, to investigate the reversibility of adsorption.

## Results and Discussion

### XPS Survey Spectra

**Figure 1:**
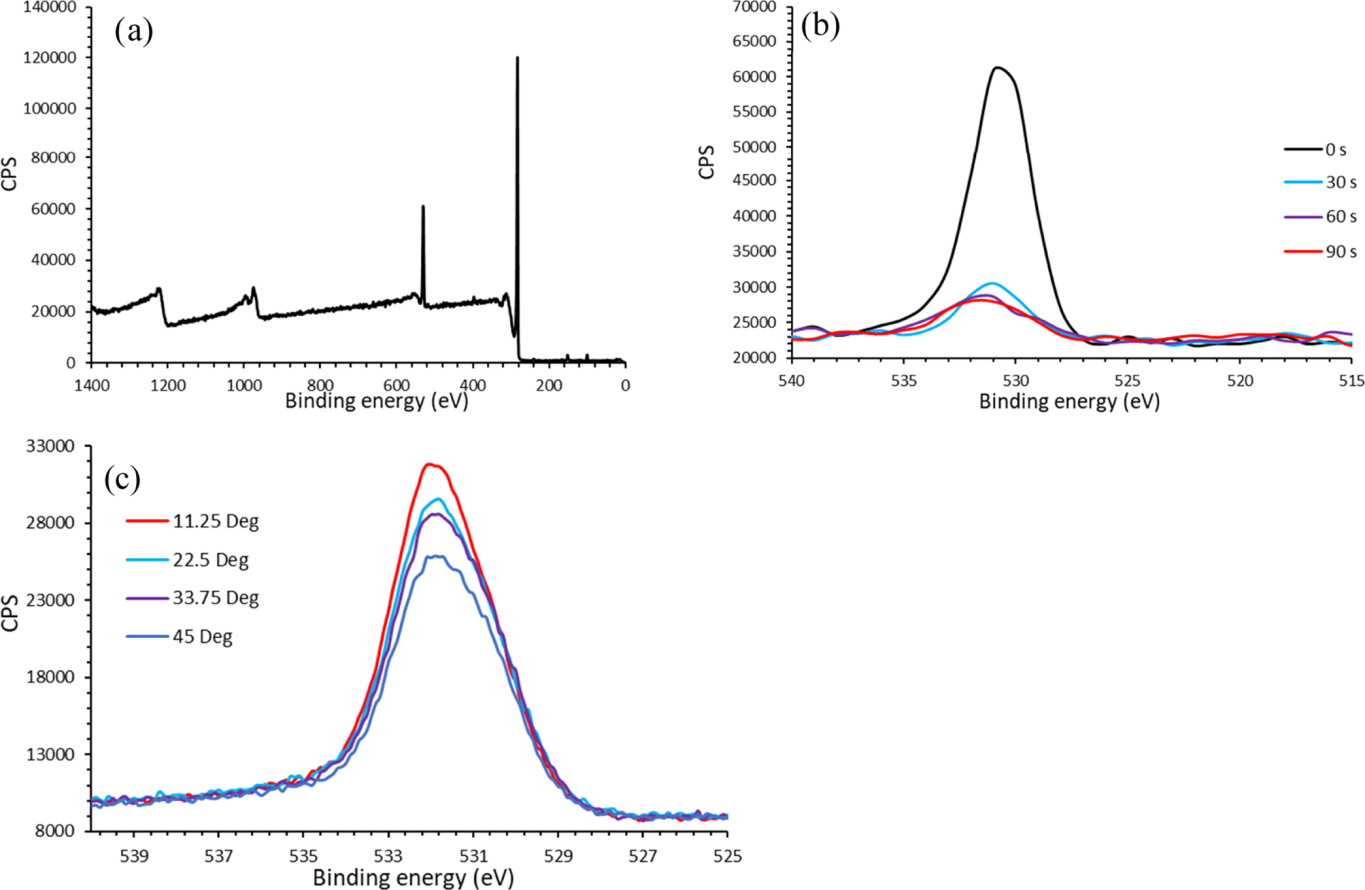
XPS spectra of a DLC film showing signal intensity (counts per second, CPS) as a function of binding energy. (a) Survey spectrum, dominated by C 1s (285 eV) and O 1s (531 eV) peaks; the composition based on the area of each peak suggests 87.7% C, 9.7% O and 2.6% of other elements including N and Si. (b) O 1s peak as a function of ion-beam etching time; a significant drop in the peak is seen after 30 s etching, suggesting the oxygen signal is predominantly from the surface. (c) Angle-resolved XPS showing a decrease in the O 1s signal intensity with increasing beam angle (angle between the beam and line parallel to the surface).

The spectra of the silicon-supported DLC layer (200 Å), shown in Figure 1a, are dominated by the C 1s peak at approximately 285 eV, as expected. Also present is the O 1s peak at 531 eV, suggesting the surface is oxygenated. Ion beam depth profiling (Figure 1b) to a depth of approximately 30 s of etching, (corresponding to roughly 1 nm) shows the intensity of the oxygen signal is significantly reduced, suggesting the O 1s peak is from the material at the surface. The energy corresponds to an oxygen environment such as –OH, as discussed in more detail below.

Angle-resolved XPS (Figure 1c) shows a drop in O signal intensity as a function of the angle between the incoming beam and the surface. As lower angles provide more surface sensitivity due to the reduced sampling depth, this is in agreement with the depth profiling results. Hence, both XPS approaches suggest the surface is oxygen-rich compared to the bulk of the film.

### XPS C 1s Peak Fitting

**Figure 2:**
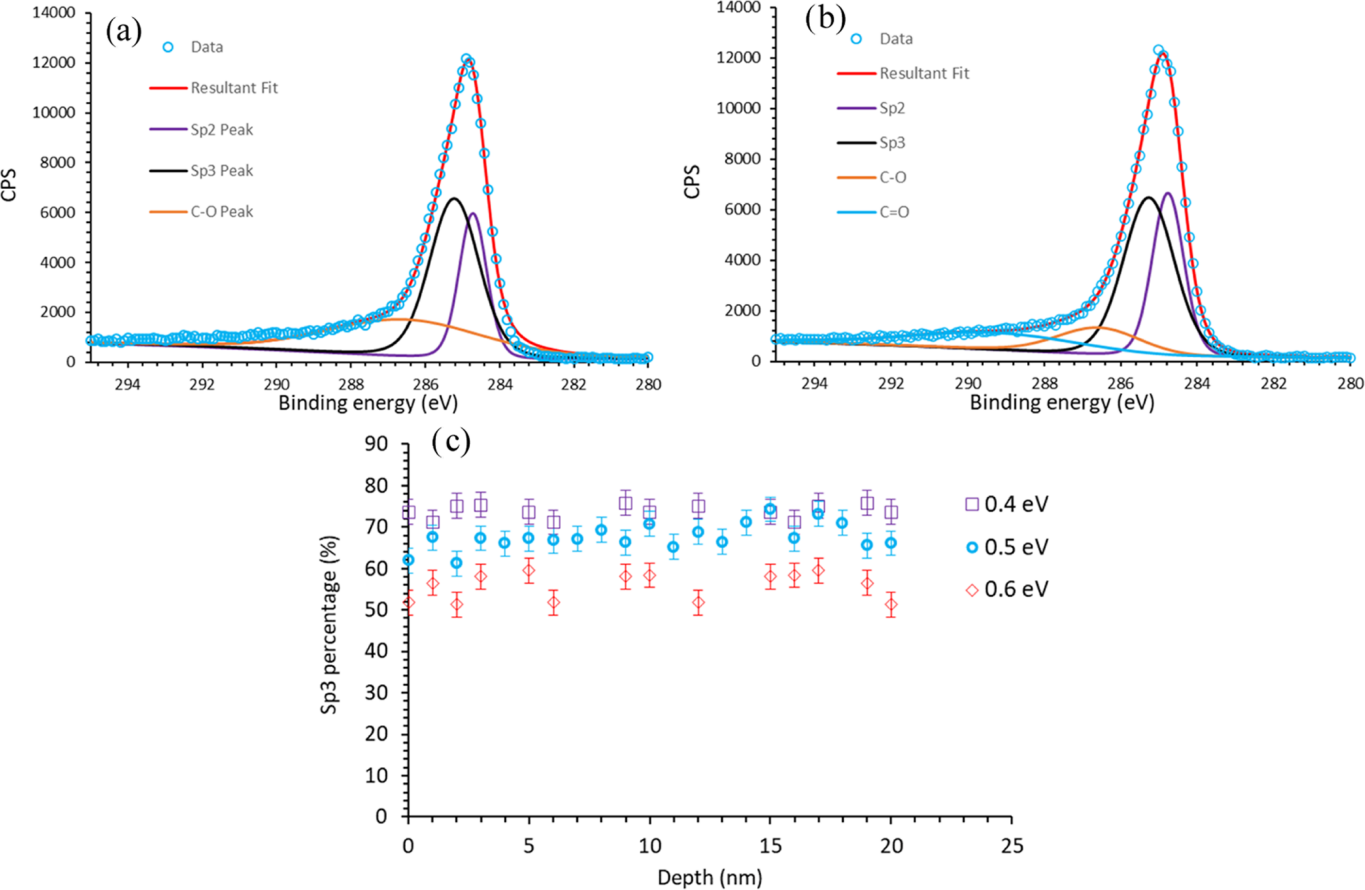
(a) XPS C 1s spectra fitted with 3 peaks, corresponding to sp^2^, sp^3^, and C–O chemical environments. (b) XPS C 1s spectra fitted with 4 peaks; when the additional peak corresponding to the CO chemcal environments is added, the overall peak captures the shape of the experimental data. (c) The percentage of sp^3^ carbon as a function of depth; the legend refers to the gap constraint used between sp^2^ and sp^3^ peaks in the fitting.

XPS can help quantify the proportion of sp^3^/sp^2^ carbon in the DLC layer. It has been reported in the literature that the C 1s peaks of pure diamond (sp^3^) and graphite (sp^2^) are at 287 and 284 eV, respectively. This difference arises from the different hybridization, i.e., the different chemical environments of the C 1s electrons in the diamond and graphite. Therefore, the shape of the C 1s feature from a DLC layer is a result of the sum of the two different hybridization states. Given the hybrid, mixed nature of the DLC, the chemical environments of the sp^2^ and sp^3^ carbons are not expected to be the same as those of pure graphite or diamond. Leung et al.^32^ reported a separation of 0.5 eV between the sp^2^ and sp^3^ chemical environments. Therefore, in our fitting procedure, the position of the sp^2^ peak was constrained to be 0.5 eV lower in energy than the sp^3^ peak. Furthermore, Merel et al.^20^ attributed an additional peak at 285.6 eV, 1.3 eV higher in energy than that of the sp^3^ peak, to C bonded to O (C–O) and Lomon et al.^21^ an additional peak for C=O. Therefore, an additional peak, centered around 1.3 eV above the sp^3^ peak, was included to account for C–O. From the resultant fitting of the experimental data (Figure 2a), the C–O peak is very broad and does not capture the shoulder of the peak above 289 eV. Therefore, similar to Lomon et al. an additional peak is added for a C=O contribution, which shows good agreement with the data (Figure 2b). The optimization program also gave the full width half-maximum (fwhm) of the fitted peaks to be 1.5 eV (sp^3^) and 0.97 eV (sp^2^), which are in good agreement with the values reported in the literature for DLC films.^32^ Similarly, the fwhm of diamond and graphite peaks have also been reported to be 1.1 and 0.98 eV, respectively.^20,32^

Depth profiling XPS measurements enabled us to characterize the proportion of sp^2^/sp^3^ as a function of depth into the DLC film (Figure 2c). This figure shows a composition of 60–70% sp^3^ and no significant variation with depth. It is important to note, due to the featureless nature of the peaks, that the composition of sp^2^/sp^3^ is significantly dependent on the relative peak position constraint (i.e., the 0.5 eV gap between sp^2^ and sp^3^ peaks). Changing this constraint to 0.4 or 0.6 eV significantly changes the composition of sp^2^/sp^3^. This provides an estimate of the possible errors in the composition, which is 10%. Note that the depth is estimated based on the etching time, and there is likely to be a significant error in the absolute value of the depth. However, as there is no significant variation in the hybridization state composition as a function of etching time, the accuracy of the conversion from etching time to etching depth is not important in this particular case.

A similar XPS study was carried out on the DLC film used in the QCM, obtained commercially. A slightly lower proportion of sp^3^ is found, 50–60%, compared to the other sample. However, angle resolved XPS shows a similar trend in oxygen signal intensity as a function of angle, suggesting that this sample is also oxygenated at the surface. Therefore, the surface chemistry of the two films is expected to be similar in nature.

### TOF-SIMS

TOF-SIMS measurements were carried out to further understand the surface and bulk chemical composition and functionality. TOF-SIMS involves removing species from the surface and measuring the intensity of different mass-to-charge ratio signals as a function of time. The aim is to gain an understanding of how each species varies as a function of depth in the film. However, it is important to note that it is not a perfect layer-by-layer removal of species, as different species have different susceptibilities to the ion beam. Furthermore, the sensitivity toward each species is different, partly due to the difference in ease of charging and the sign of the ionic charge produced.

**Figure 3:**
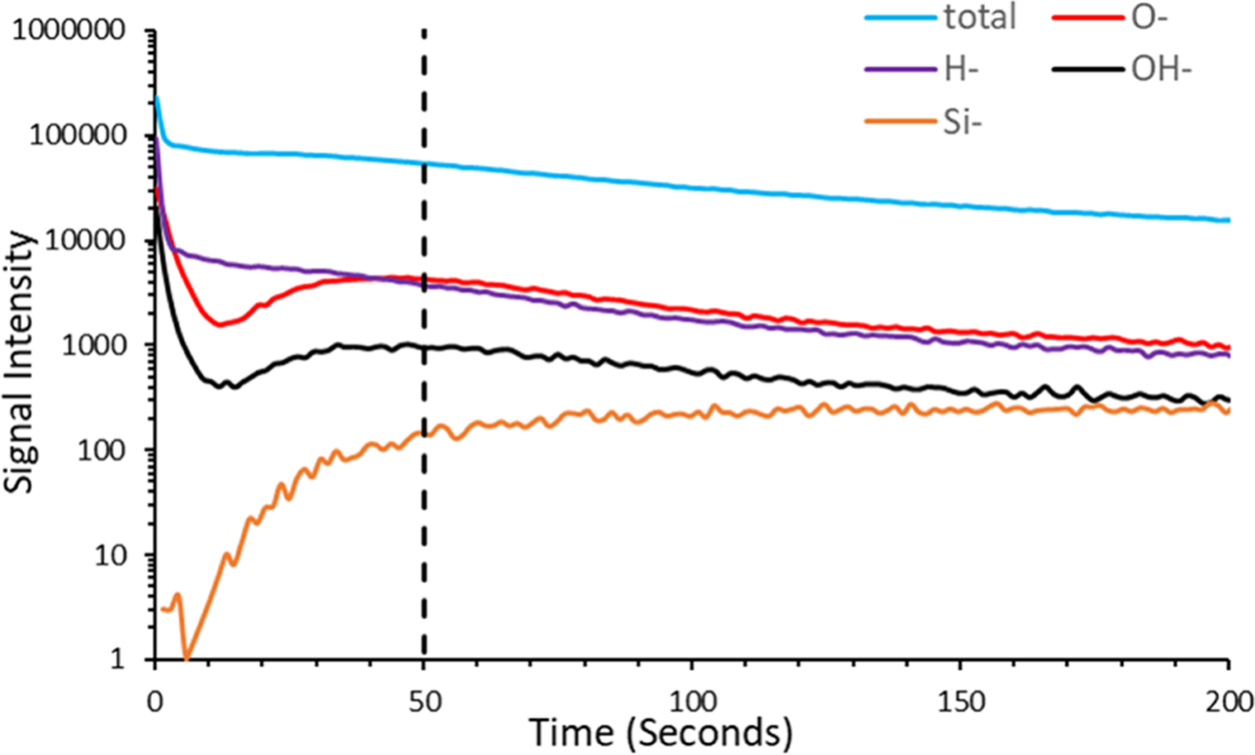
TOF-SIMS results for a silicon-supported DLC film as a function of time; a sharp drop in the oxygen and hydrogen species is seen at the beginning (<5 s), suggesting significant levels of O and H species at the surface. A vertical black dashed line is added to mark the end of the DLC film.

Figure 3 shows TOF-SIMS data for a silicon-supported DLC film. The Si signal increases with depth, before essentially reaching a plateau. This corresponds to the ion beam drilling down through the DLC layer into the silicon substrate below. Hence, we can conclude that the region up to approximately 50 s is most relevant for the DLC film.

The results in this 0–50 s region (the DLC film) initially show a significant drop in the oxygen and hydrogen signals, followed by a slight rise on entering the Si substrate. This suggests that more oxygen and hydrogen are present at the air/DLC surface than in the bulk, which is in agreement with the XPS data above. The presence of hydrogen suggests the surface of the DLC film is either hydrogenated (i.e., contains C–H) or, given the occurrence of the surface oxygen, terminated with O–H groups. The presence of an OH signal and the contact angle of 55°, suggesting a slightly hydrophilic surface, support the latter interpretation.

The rise in O and OH signals at the DLC/silicon interface suggests that SiOH groups are present on the native silicon used to prepare the DLC layer. The positions of the XPS O 1s peak arising from the DLC surface and the native silicon dioxide surface are both around 531 eV, further supporting the presence of OH at the DLC-air interface.

### GIWAXS

**Figure 4:**
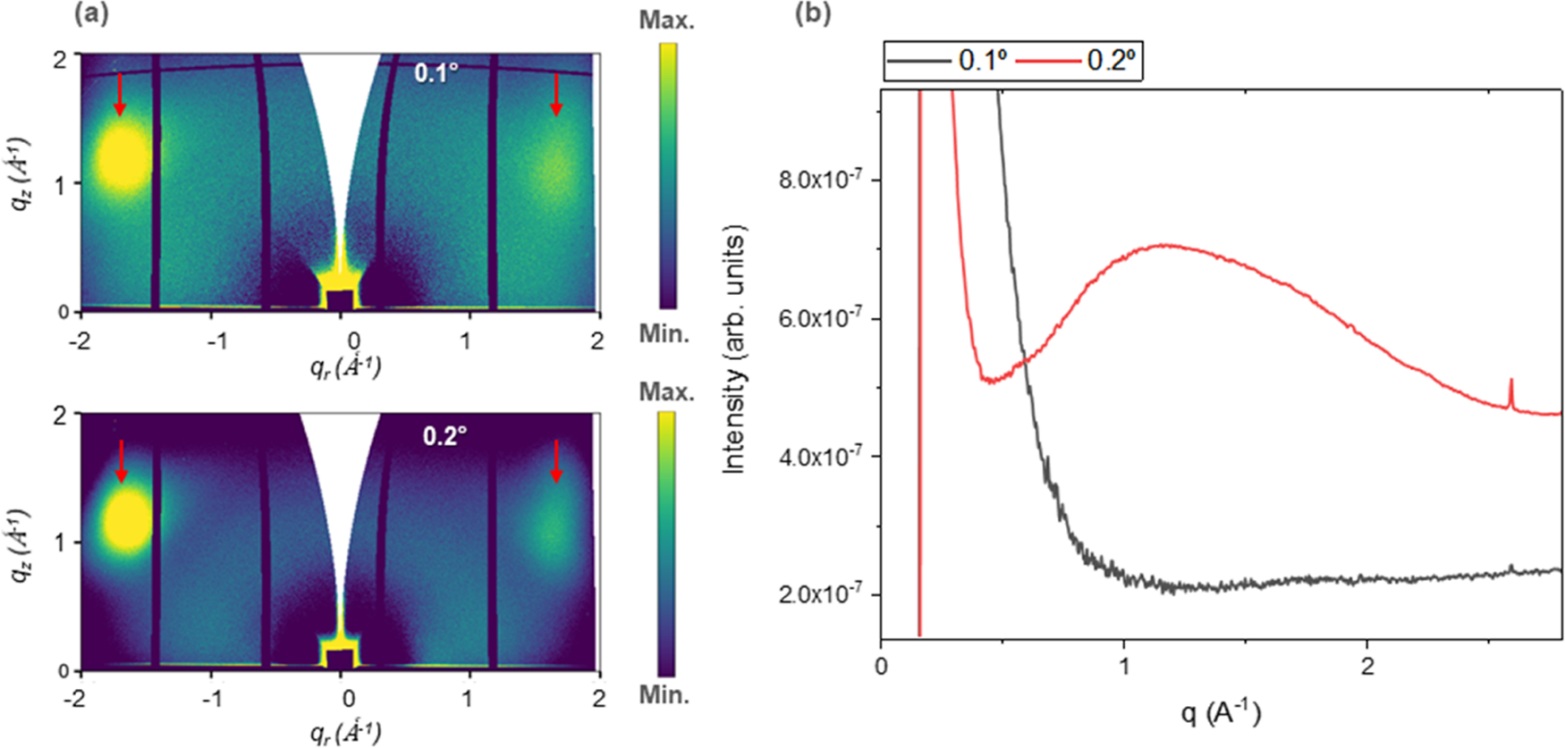
(a) GIWAXS patterns from the sample, collected at two grazing angles: 0.1 and 0.2°. Scattering features coming from the silicon are highlighted with red arrows. (b) Intensity profile obtained after an azimuthal integration of the pattern along the vertical direction of *q* (*q_z_*).

GIWAXS was used to investigate the crystallinity of the DLC layer. However, as carbon is not a strong scatterer of X-rays, and the scattering from a thin film will be small, this is a challenging experiment. Hence, a significantly thicker sample (300 Å) was prepared on a silicon wafer and measured in grazing-incidence conditions to optimize the signal. Figure 4a presents the GIWAXS profile of this DLC film. The original detector arrays were transformed into momentum transfer vector; q_z_ is the momentum transfer along the normal direction of the substrate, and *q*_r_ is the average of both momentum transfers along the substrate plane . This is a common representation for GIWAXS patterns, since it allows visualization of both vertical and horizontal orientations of the crystalline planes in a thin film. There are two main scattering features in the patterns: two peaks at both sides of the pattern (around *q_z_* ∼ 10 Å^–1^) and a wide, isotropic halo between *q* = 10 and 15 Å^–1^. The two peaks, much more intense than the halo, come from the silicon substrate. The fact that the peaks do not present the same intensity is due to this particular orientation of the silicon crystalline planes regarding the incoming beam. On the other hand, the wide, isotropic halo is attributed to the carbon layer. For a better visualization of the halo, the patterns were integrated along the vertical direction (*q_z_* axis) (Figure 4b). Illuminating the sample with an incident angle from 0.1 to 0.2°, the corresponding penetration depth for these measurements varies from a few nanometers to several microns (the whole carbon thickness). There was no evidence of crystallinity in the DLC films. This may be due to the amorphous nature of the films, but it may also be due to the poor X-ray scattering power of the carbon, so this conclusion should be treated with caution.

**Figure 5:**
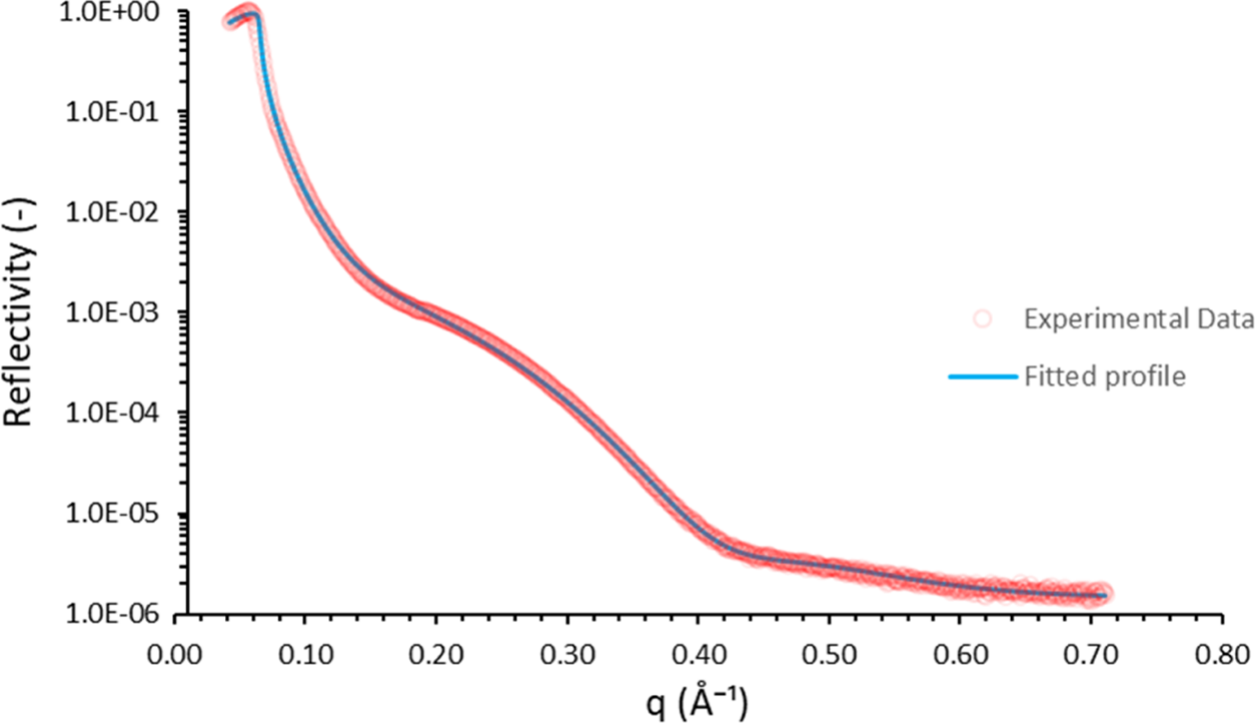
XRR profile of an approximately 28 Å thick DLC film on a silicon wafer.

**Table 1:** Fitted Parameters from the XRR Profile, Showing the Thickness, Density, and Roughness of Each Layer^b^

medium	thickness (Å)	density (g/cm^3^)	roughness (Å)
Si	∞	2.33^a^	6 ± 2
SiO_2_	18 ± 2	2.16^a^	4 ± 2
DLC	28 ± 3	2.1 ± 0.1	6 ± 3
air	∞	0	

a Value is fixed to literature value.

b This also enables the expected neutron scattering length density (SLD) to be calculated. The fitted background value is 1.5 × 10^–6^.

### XRR

An XRR study was carried out to characterize the silicon-supported DLC film and to obtain key information such as its thickness, scattering length density, and interface roughness. The data are shown in Figure 5, and the fitted parameters from the XRR profile are presented in Table 1; this particular DLC film is found to be 28 Å thick. These data were also used to constrain parameters in the NR fitting, as discussed below. As the density of the native silicon dioxide layer is a little lower than the literature value of 2.65 g/cm^3^, it is likely that the native oxide layer is somewhat porous. The density of the DLC (assuming the bulk is pure carbon) is approximately 2.05 g/cm^3^. The reported density of amorphous carbon^33,34^ is in the range 2–2.3 g/cm^3^, while the density of DLC coated films^35^ is reportedly in the range 1.09–3.15 g/cm^3^. Therefore, the density of the DLC film obtained here is within bounds of previous studies.

### Neutron Reflectivity

**Figure 6:**
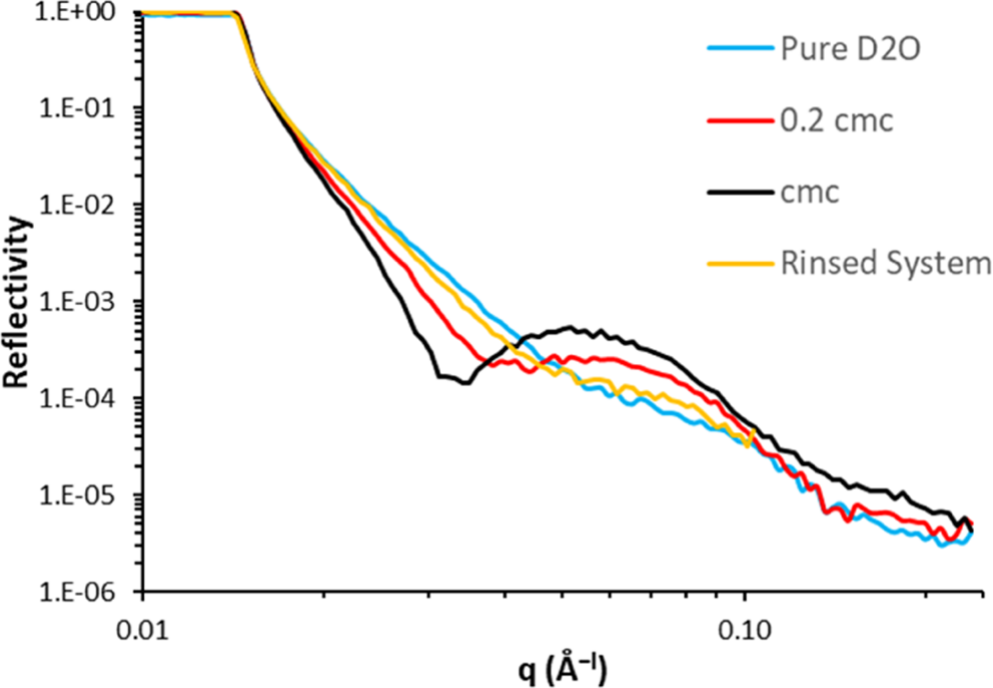
Neutron reflectivity profiles of the DLC film exposed to varying concentrations of AOT surfactant. The reflectivity profile changes significantly with AOT concentration, suggesting an adsorbed layer forms.

Neutron Reflectivity data from the DLC surface exposed to pure D_2_O and AOT concentrations of 0.2 and 1.0 CMC (critical micelle concentration, approximately 2.5 mM) are presented in Figure 6. The bare substrate exhibits the profile expected from a D_2_O contrast with a silicon substrate, with a region of total reflection and a critical angle at low *q*. Importantly, the figure shows a significant change in the reflectivity profile (compared to the bare surface) on exposure to the surfactant, indicative of an adsorbed AOT layer on the surface. The profile continues to change as the concentration increases from 0.2 CMC to 1.0 CMC. Interestingly, after the system is rinsed with pure water and refilled with D_2_O, the reflectivity profile returns close to, but not exactly the same as, that obtained initially with pure D_2_O. This suggests that the layer of AOT surfactant is mainly reversibly physisorbed.

### Base Substrate

**Figure 7:**
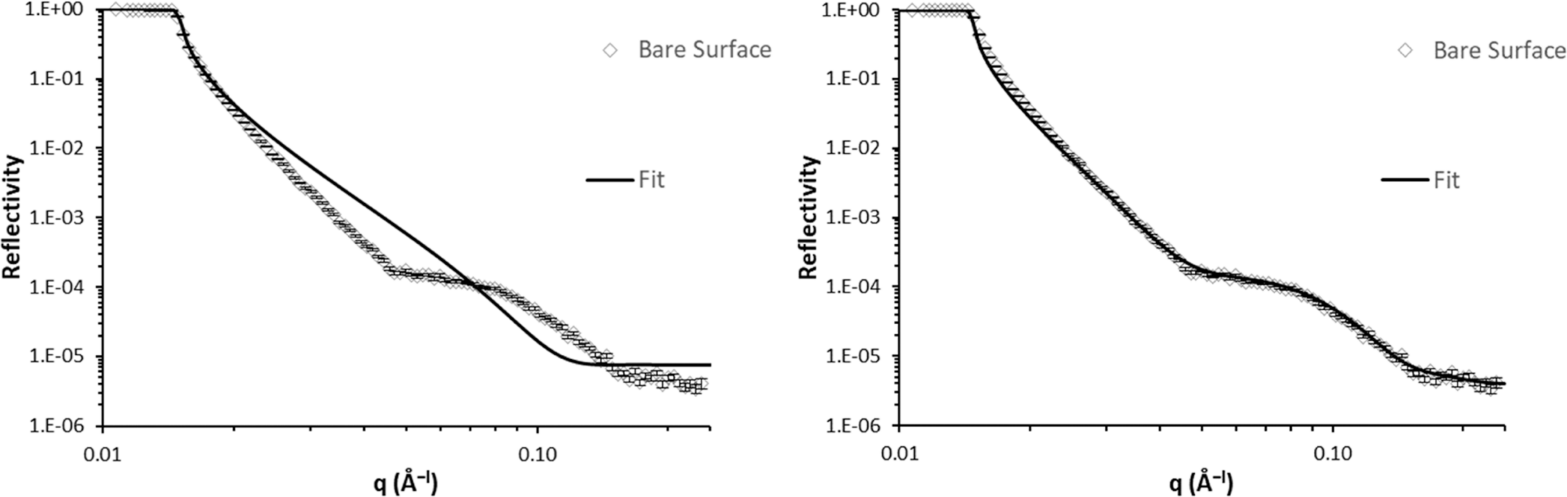
Neutron reflectivity profile of the bare surface in pure D_2_O. (Left) The reflectivity profile is fitted with a single-layer model for the DLC, similar to the model used for the XRR study (Table 1); the parameters are constrained within ±40% of the values obtained from XRR, but no reasonable fit is obtained. (Right) An additional layer of low SLD is added to the surface of the DLC film, and all other parameters are constrained within ±5% of the parameters obtained from XRR; this captures the profile of the experimental data very well.

Using the XRR data presented in Figure 5 and the corresponding fitted parameters in Table 1, the neutron reflectivity profile of the DLC film exposed to pure D_2_O can be fitted. The XRR profile provides a very good estimate of the thickness and SLD value of each layer, which helps to constrain the fitting of the NR data. It was found that including an additional layer (labeled here as a ‘DLC-surface’ layer), with a low SLD and thickness 5–6 Å, on the DLC was necessary to obtain a good fit, as shown in Figure 7. The low SLD of this DLC-surface layer could indicate a region of hydrogenated material on the DLC or as part of the DLC close to the surface. This is consistent with the TOF-SIMS results, where a significant amount of hydrogen is present at the surface (probably as OH groups). The SLD value seen here is lower than the value of 2.6 × 10^–6^ Å^–2^ reported in the literature for a hydrogenated DLC film^10^ with composition CH_0.78_, suggesting the composition of the hydrogen in the DLC-surface layer of this sample is higher than CH_0.78_. There is no indication of this low SLD region at the surface in the XRR profile, due to the low relative contrast. The parameters from the NR fitting are in Table 2.

**Table 2:** Fitted Parameters Obtained from the Bare Surface NR Profiles

	fitted parameter		
medium	thickness (Å)	SLD × 10^–6^ (Å^–2^)	roughness (Å)
Si	∞	2.07^a^	8 ± 4
SiO_2_	19 ± 3	3.41^a^	1 ± 0.5
DLC	32 ± 3	6.2 ± 0.2	3 ± 2
DLC-surface layer	5 ± 1	1.8 ± 0.4	2 ± 2

a Values are fixed to expected values from the literature. The fitted background value is 3.5 × 10^–6^.

### Adsorbed Surfactant Layer

There are a number of possible structures for the adsorbed surfactant layer, one of which is a ‘side’ adsorption, where the molecules lie flat on the surface; this layer can be modeled most simply as a single, rather thin, layer of uniform SLD. Another possibility is a bilayer: Since the DLC surface is hydrophilic (or has a sufficient number of hydrophilic groups, such as –OH), the polar ‘heads’ of the AOT might be expected to be on the surface. However, a head down-tail up structure is unlikely, due to the hydrophobic nature of the hydrocarbon tail; it is energetically unfavorable for the tail to mix with the bulk D_2_O. An adsorbed bilayer would allow the polar ‘heads’ to interact with the surface, without the tails having to mix with the bulk D_2_O, as the head groups of the second layer of AOT extend into the bulk solution.

**Figure 8:**
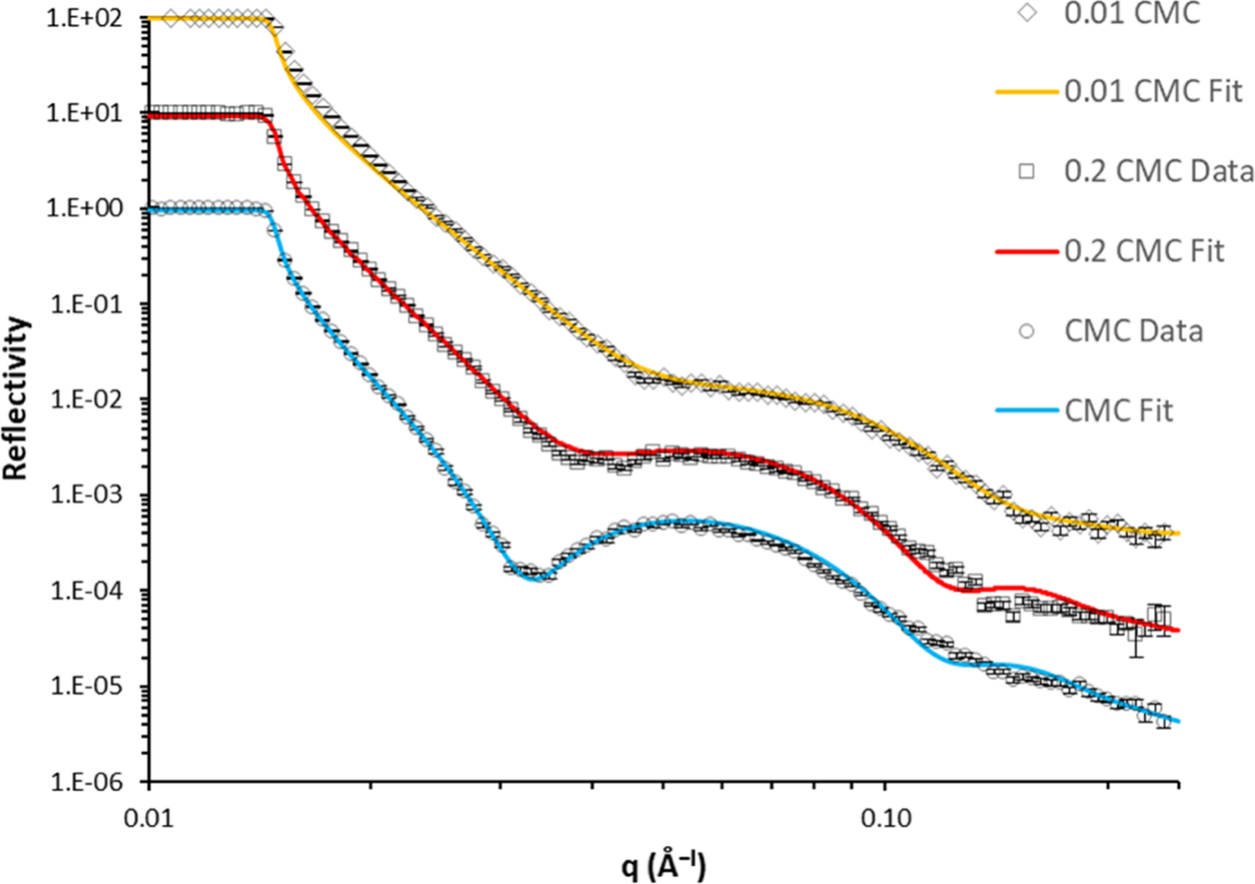
Neutron reflectivity profiles of a DLC film exposed to varying AOT concentrations and the corresponding fits obtained with a single-layer model.

The SLD of a single layer^36^ of NaAOT is 0.65 × 10^–6^ Å^–2^. With the fitting parameters of the Si, SiO_2_, DLC, and DLC-surface layers fixed at the values obtained from fitting the bare surface (Table 2), the experimental data with adsorbed AOT were fitted using different models, including those described above. The parameters were constrained to be in sensible physical limits, based on the molecular sizes. Using a simple single-layer model for the AOT shows a good fit to the data (Figure 8). The fitted parameters are presented in Table 3.

**Table 3:** Parameters Obtained from the NR Fitting: Parameters Corresponding to the DLC Film and Bulk D_2_O were Fixed at the Values Given in Table 2, So the Parameters Corresponding to the AOT Adsorbed Layer could be Fitted^a^

medium	thickness (Å)	solvation (%)	roughness (Å)
0.01 CMC	15 ± 7	85 ± 15	7 ± 6
0.2 CMC	22 ± 3	68 ± 8	8 ± 2
1 CMC	27 ± 2	53 ± 5	7 ± 2

a The fitted background value is 4.0 × 10^–6^.

The extended chain length of a monolayer of the AOT is reportedly approximately 18 Å.^37^ The single-block model indicates a layer thicker than this (27 Å), suggesting there is a bilayer of AOT. An adsorbed bilayer appears reasonable, for the reasons discussed above. The overall layer thickness obtained from the fitting is somewhat less than the 36 Å expected for two AOT molecules end to end; hence, it appears that the tails from the AOT molecules are interdigitated and/or tilted at the surface. The fitted solvation amounts, 53–85%, suggest significant solvent is present in the adsorbed layer.

The AOT head and tail groups have different SLDs, which can be estimated based on the scattering length of each atom and estimates of the corresponding molecular volume. Allen et al.^36^ reported the SLD of the protonated hydrocarbon tail as −0.42 × 10^–6^ Å^–2^ and that of the headgroup as 4.25 × 10^–6^ Å^–2^. Others have made similar but slightly different estimates.^38^ To reflect this, more complex models, such as a three-layer model (head-tail-head), where the AOT head and hydrocarbon tail regions are treated separately, were also considered. However, when the SLD’s of the layers are fixed at the values reported in the literature for the heads and tails (assuming no mixing), no reasonable fit of the data can be achieved. This is true for all parameter space spanned over physically reasonable layer thickness, roughness, and hydration, as demonstrated in the Supporting Information. The unmixing of heads and tails result in a stark contrast, but allowing some penetration of the hydrocarbon tail into the head region improves the fit significantly.

**Figure 9:**
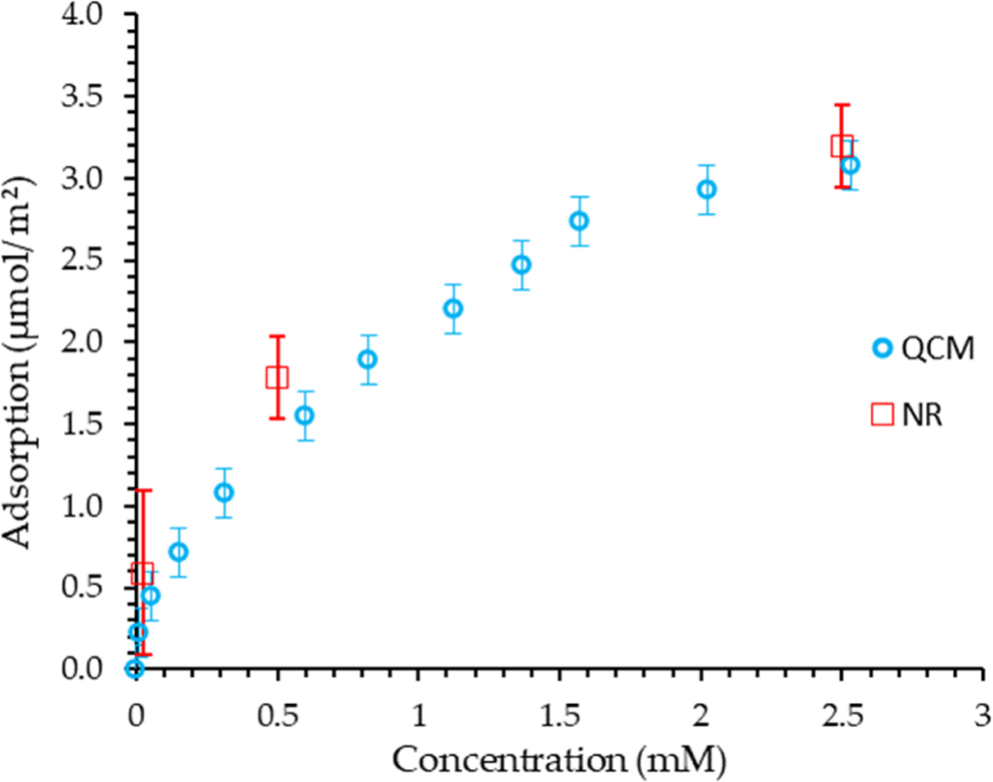
Adsorption isotherm of the AOT surfactant onto the DLC film from water at 20 °C, measured with the QCM method and the neutron reflectivity profiles.

From the solvation and thickness of the adsorbed layer, the adsorbed amount can be calculated.^37^ The adsorbed amounts of AOT as a function of solution concentration from NR fitting are shown in Figure 9. This gives an area per two molecules (i.e., per bilayer) of 102 Å^2^ at the CMC. However, it is important to note that the adsorbed amount and the area per molecule calculated with this method represent the average over the entire surface. Specular neutron reflection only provides the variation in the average SLD in the direction normal to the surface, with no information about the in-plane structure. Hence, there is no possibility of differentiating between a uniformly adsorbed layer and patches of more densely adsorbed species, interspersed with patches without adsorption. These two possibilities can be differentiated using off-specular neutron reflection; however, no significant off-specular signals were seen in this work, suggesting a reasonably uniform adsorbed layer.

### QCM—Adsorption

The adsorption isotherm of AOT from water onto the DLC was also obtained using the QCM technique, as shown in Figure 9. The QCM data have been analyzed using the Sauerbrey equation, which assumes that all the mass changes are attributed to the surfactant. Acoustically bound solvent prior to the surfactant addition that may be lost on adsorption and water bound to the surfactant when adsorbed have been neglected in this calculation. The adsorption found from NR is approximately 5–10% higher than that measured with the QCM method. This discrepancy may have a number of origins, including the challenges of the experiments. It is possible that the ‘solvent-replacement’ nature of the QCM may give an effect; i.e., the QCM measures the mass difference between the adsorbed AOT and the water molecules that have been replaced on the surface by the AOT, although this depends on how well acoustically coupled the solvent is to the substrate. Another source of discrepancy may be differences in the surface roughness of the DLC film and the QCM substrates. There may also be differences in the precise composition of the DLC layers used for NR and QCM measurements, as described in the Experimental section.

If we assume there is an adsorbed bilayer, at the CMC, the area calculated per two molecules of AOT from the QCM and NR data are 108 and 102 Å^2^, respectively. A similar study in the literature carried out at the water-alumina interface reported an approximate area of 51 Å^2^ per two molecules (per bilayer) at the CMC.^36−38^ Another similar study on the adsorption of AOT on self-assembled monolayers of octadecyltrichlorosilane (OTS) on silicon reported a somewhat more diffuse layer, with an approximate area of 80 Å^2^ per bilayer.^39^ Therefore, we conclude that the AOT bilayer at the water-DLC interface is more diffuse, with approximately half the number density of adsorbed molecules compared to the bilayer adsorbed at the alumina-water interface. This may be a consequence of a lower number density of -OH groups at the DLC surface, which is possible if not every surface carbon atom is bound to an -OH group. However, the number density of -OH groups at the DLC surface is unknown at present.

### Conclusions

We have presented an in-depth characterization of DLC films using a range of different techniques to understand the structure, chemistry and functionality both in the bulk and at the surface. We have successfully shown:

- This DLC is a mixture of both sp^2^ and sp^3^ carbons, with approximately 60–70% sp^3^ carbon. The hybridization composition does not show any significant changes with depth.
- There is no evidence of any crystallinity in this DLC structure.
- There is significant evidence from multiple techniques that suggest large amounts of oxygen and hydrogen are present at or near the DLC surface, which is tentatively attributed to surface –OH groups that make the surface hydrophilic.
- AOT surfactant adsorbs as a bilayer on the DLC film, but with a somewhat lower number density than on related substrates, such as alumina.

## Supporting Information Available

Brief discussion of the alternative models used for analysis of neutron reflectivity data with an adsorbed AOT layer (PDF)

## Notes

For the purpose of open access, the author has applied a Creative Commons Attribution (CC BY) license to any Author Accepted Manuscript version arising from this submission.
