# Supplementary material for: Diamond-Like Carbon: A Surface for Extreme, High-Wear Environments
Source: Langmuir. 2023 Dec 19;40(1):52–61. doi: 10.1021/acs.langmuir.3c01438 (PMC10786025; doi:10.1021/acs.langmuir.3c01438)
Supplement: Supplementary file 1 — la3c01438_si_001.pdf [file la3c01438_si_001.pdf]

Diamond-Like Carbon: A Surface for Extreme, High-Wear Environments

N. Sharifi[1], H. Smith[1], D. Madden[1], T. Kehoe[1], G. Wu[2], L. Yang[2], R. J. L. Welbourn[3], E. G. Fernandez[4,5] S. M. Clarke[1*]

[1]Institute for Energy and Environmental Flows and Yusuf Hamied Department of Chemistry, University of Cambridge, Cambridge, CB2 1EW, UK.

[2] Institute of Functional Surfaces, School of Mechanical Engineering, University of Leeds, Leeds, LS2 9JT, UK

[3]ISIS Neutron & Muon Source, Rutherford Appleton Laboratory, STFC, Chilton, Didcot, Oxon, OX11 0QX, UK

[4]XMaS/BM28-ESRF, 71 Avenue Des Martyrs, F-38043 Grenoble Cedex, France

[5]Department of Physics, University of Warwick, Gibbet Hill Road, Coventry CV4 7AL, U.K.

*Address correspondence to this author: sc10015@cam.ac.uk

Supplementary information

Neutron Reflection Fitting Examples

Fitting the AOT Layer

A number of different models were used to fit the 1CMC data of AOT adsorbed on the DLC surface. These models included a single layer of uniform SLD (i.e. 1 layer model) and different configurations of 2 layer model (i.e. head-tail or tail-head orientation or completely unconstrained 2 layer model) and a 3-layer model corresponding to a bilayer of head-tail-head. The single-layer model is described in the main paper. Here the 3-layer model is presented as an example of fits carried out to investigate other models. The fit to experimental data of both models is presented in Fig. S1 and the corresponding fitted parameters for the three-layers model is presented in Table S1. Changing the model from a single layer to three-layers results in a worse fit to experimental data. The SLDs of the tail and head are fixed to -0.42×10^{-6} Å^{-2} and 4.25×10^{-6} Å^{-2} respectively. The solvation, thickness and roughness of each layer was fitted. However, it is important to note, that these parameters are constrained within the maximum range possible for these to be physically possible. For example, the head-group has a thickness of 6 Å and the tail group around 20 Å. We have therefore constrained the thickness of the head group to be in the range 3-12 Å thick, rather than let it freely vary to any value. The single-layer model suggests that this layer has a significant amount of water entrained in the adsorbed layer, it is likely that the AOT has adsorbed in patches. For the three-layer model, the solvation amount of each layer was constrained to be the same and the results are presented in Table S1, the solvation amount is similar to the one-layer model which provides further confidence in the one-layer model.

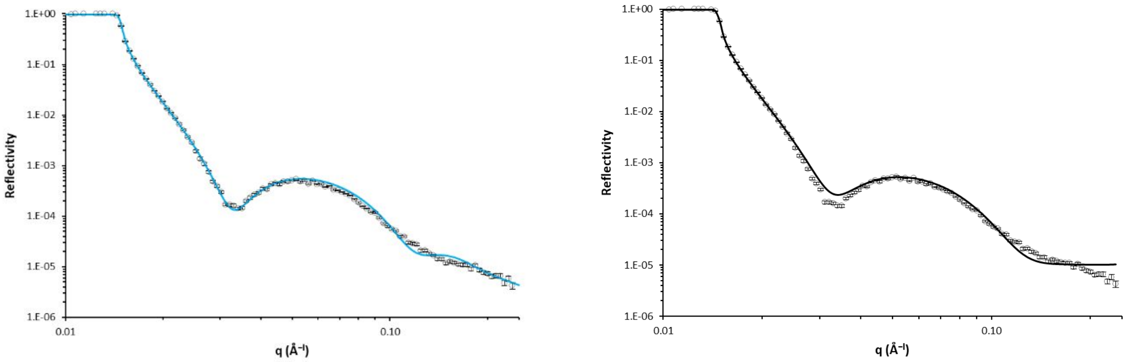

Figure S1. Log Models used to fit AOT adsorbed onto the DLC surface at 1 CMC. left) 1-layer model of uniform SLD. right) 3-layer model for bilayers.

Table S1. Fitted parameters from the three-layer model where the solvation of all three layers is constrained to be the same. The fitted background value is 4.0×10^{-6}.

Medium	Thickness (Å)	Solvation (%)	Roughness (Å)
Head	5.2	54	0.7
Tail	19.9	54	3.2
Head	5.2	54	4.2

Further analysis, where the solvation of each layer is allowed to vary independently (Table S2), results in a similar 'goodness of fit' to the experimental data, however, gives physically unreasonable results where the solvation amount of the hydrophobic tail is significantly higher than the head group. This suggests the model is overfitting the data hence it is not sensitive to the individual solvation amounts.

Table S2. Fitted parameters from the three-layers model where the three-layers are enabled to have different solvation amounts. The fitted background value is 4.0×10^{-6}.

Medium	Thickness (Å)	Solvation (%)	Roughness (Å)
Head	6.0	0.0	3.1
Tail	17.2	61.0	4.1
Head	6.0	0.0	3.6
